# Low dose naloxone for pruritus in systemic sclerosis: Case series and literature review

**DOI:** 10.1097/MD.0000000000028653

**Published:** 2022-01-28

**Authors:** Katya Meridor, Joshua Berookhim, Yair Levy

**Affiliations:** Department of Internal Medicine, Meir Medical Center, Israel; affiliated with the Sackler Faculty of Medicine, Tel Aviv University, Tel-Aviv, Israel.

**Keywords:** naloxone, opiate antagonists, pruritus, systemic sclerosis

## Abstract

**Rationale:**

Pruritus is a common symptom in patients with systemic sclerosis and has a tremendous effect on the quality of life. Nevertheless, current therapeutic options are limited. The pathogenesis of pruritus in systemic sclerosis is not completely understood; however, opiate-mediated neurotransmission has been postulated to be involved.

**Patient concerns and diagnosis:**

We describe 4 female patients with systemic sclerosis suffering from severe pruritus, with an average 5D-itch score of 22.75.

**Intervention and outcomes:**

Low-dose oral naloxone was initiated, followed by a significant improvement in the level of pruritus, reaching an average 5D-itch score of 7.5, after 6 and 12 months of treatment. None of the patients experienced side effects.

**Lessons:**

Low-dose naloxone plays an important role in the management of pruritus in systemic sclerosis.

## 1 Introduction

Systemic sclerosis (SSc) is an autoimmune connective tissue disease characterized by abnormal fibrosis of the skin and internal organs. The peak age of onset is between 30 and 50 years, with a female:male ratio of 5:1.^[1,2]^ The organs that are commonly involved include the skin, lungs, heart, kidneys, and the gastrointestinal tract. Cutaneous features include thickening of the skin that can transition to sclerodactyly with the obliteration of hair follicles and sweat glands. Raynaud phenomenon, which is a reversible vasospasm due to functional changes in the digital arteries of the hands and feet, is also present in most patients with SSc.^[3,4]^ Frequent complications of SSc include esophageal dysmotility, hypertension due to renal involvement, and interstitial lung disease.^[5–7]^ Pruritus is a common symptom of SSc and has been shown to affect many important aspects of an individual's quality of life.^[8–10]^ Pruritus is associated with sleep disruption, overall disability, depression, and fatigue. Many patients also report scratching until they bleed.^[11,12]^

The Canadian Scleroderma Research Group reported that pruritus was found in 43% of patients with SSc, was more common in early disease, and patients with pruritus had more severe skin disease and greater gastrointestinal tract involvement.^[8,13]^ Théréné et al^[14]^ reported that the prevalence of pruritus was as high as 62% in patients with SSc patients. Despite its high prevalence, there are no clear guidelines for the management of pruritus in SSc patients.

The pathogenesis of pruritus in SSc is still not completely understood and is not specifically associated with a particular immunological profile.^[15]^ Various pruritogens, including histamine, lysophosphatidic acid, prostaglandin E2, endothelin-1, and neuropeptides from unmyelinated nerve endings, have all been linked to pruritus in SSc.^[16]^ Furthermore, opioid receptors have been implicated in the pathogenesis of itch. It has been hypothesized that proinflammatory and profibrogenic cytokines may be modified through the modulation of opioid receptors in SSc, similar to hypertrophic scaring.^[16–19]^ Currently, the therapeutic options for pruritus in SSc include anti-histamines, gabapentin, and phototherapy; however, many patients are nonresponsive to these treatments.^[16]^ Treating pruritus in SSc with opiate antagonists has been reported in a single small case series previously^[20]^ with positive results.

In this case series, we report our experience with low-dose naloxone (an opioid receptor antagonist) in 4 of our patients with SSc and pruritus and review the relevant literature.

## 2 Patients and methods

We report 4 female patients with SSc who had notable pruritus and were followed up in our outpatient rheumatology clinic in Meir Medical Center. Demographic information was obtained from the medical records and self-reports. The study physicians obtained the patients’ medical background, disease clinical and serological characteristics, use of medications, and laboratory tests. The pruritus level was assessed using the 5D-itch scale.^[21]^ The 5D-itch scale is a multidimensional method to measure itching, which has been validated in patients with chronic pruritus to detect changes over time. The scale considers the duration, degree, direction, disability, and distribution. The 5D-itch scale score ranged from 5 (no pruritus) to 25 (most severe pruritus). At the time of recruitment, all patients had pruritus scores >20 (average score of 22.75). Each patient was started on 4.5 mg of oral naloxone once daily, and their degree of pruritus was assessed using the 5D-itch scale after a week, 6, and 12 months. All the patients were monitored for side effects during the treatment period.

## 3 Results

Four female patients with SSc aged between 31 and 74 years were included. Three patients were diagnosed with diffuse cutaneous SSc and 1 patient had limited cutaneous SSc. Each patient tested positive for one or more serological markers (antinuclear antibodies, anti-topoisomerase I/ anti-scl-70, and anti-RNA polymerase III). All had Raynaud phenomenon with mild to moderate lung involvement. Each of these patients had complaints of severe pruritus, with an average score of 22.75 on the 5D-itch scale before treatment. All patients were started on oral naloxone (4.5 mg) once a day. Within 1 week all patients reported drastic improvement in the level of pruritus, with a decrease in the average 5D-itch scale to 12.5. The level of pruritus was then measured again using the 5D-itch scale 6 months and 1 year after the initiation of low-dose naloxone. The average score was 7.5 after 6 and 12 months of treatment, with complete resolution of itching in 1 patient. There was an average reduction in pruritus of 15.25 points with a *P*-value of .004. No side effects were observed with naloxone at this dose. All 4 patients were treated continuously for the first year. After 2 to 3 years, pruritus subsided in all our patients; therefore, the treatment was stopped.

## 4 Discussion

Pruritus is one of the most common and bothersome features of SSc, present in up to two-thirds of patients. Unfortunately, currently available treatment options seem to have limited and unsatisfactory effects in controlling pruritus. Several studies have shown that pruritus significantly affects quality of life and, importantly, it has been shown that the effect of pain severity on quality of life diminishes as itch severity increases.^[10]^

The pathogenesis of pruritus in these patients is unclear, but it is believed to involve opiate-mediated neurotransmission, both centrally and peripherally (through opioid receptors on different skin cell types).^[22]^ Moreover, low-dose opiate antagonists are thought to act via multifactorial modulation of the inflammatory system.^[23,24]^ This provides a rationale for treating pruritus in patients with SSc using opiate antagonists.

The beneficial effects of opiate antagonists on pruritus have been demonstrated in other conditions such as primary biliary cirrhosis and cholestatic pruritus,^[25]^ and they have been shown to improve pain and quality of life in Crohn disease^[26]^ and in multiple sclerosis,^[27]^ but the data in SSc are sparse.

To the best of our knowledge, the only report thus far on the use of opiate antagonists for pruritus in SSc was by Frech et al.^[20]^ The investigators used low-dose naltrexone hydrochloride, a pharmaceutical similar to naloxone, with significant improvement in pruritus in 3 patients with systemic sclerosis. However, in this study a 10-point faces scale was used to assess pruritus, a tool validated for the assessment of pain, mostly in children.

Our study is the second to examine the use of opiate antagonists for pruritus in patients with SSc. We used the 5D-itch scale, a multidimensional questionnaire designed to be useful as an outcome measure in clinical trials, to assess pruritus in 4 women with SSc and severe pruritus before and after treatment with naloxone. We observed a dramatic reduction in pruritus in all 4 patients within a week and a later significant and lasting effect within 6 to 12 months of treatment (average reduction of 5D-itch scale of 15.25 points, *P*-value = .004). All patients also reported fewer disturbances and improvements in sleep, leisure/social activities, housework/errands, and work/school.

Since there is no cure for systemic sclerosis, and due to the tremendous impact of pruritus on patients’ quality of life, it is of paramount importance to create new targeted approaches to pruritus management. We believe that low-dose naloxone is an effective therapy for refractory itching in patients with SSc. This drug is available, inexpensive, and has a low risk of side effects. Larger, double-blind, randomized controlled trials are needed to determine the optimal type of drug, exact dose, and duration of treatment.

## 5 Conclusions

In this case series, we report 4 patients with systemic sclerosis who showed a significant improvement in pruritus with low-dose naloxone. We suggest that low-dose naloxone is an effective treatment and should be considered in the management of pruritus in SSc patients.

## Author contributions

All authors read and agreed to the published version of the manuscript.

**Conceptualization:** Yair Levy.

**Investigation:** Joshua Berookhim.

**Supervision:** Yair Levy.

**Writing – original draft:** Katya Meridor, Joshua Berookhim.

**Writing – review & editing:** Katya Meridor.

## References

[1] Mayes MD Lacey JV Jr Beebe-Dimmer J. Prevalence, incidence, survival, and disease characteristics of systemic sclerosis in a large US population. Arthritis Rheum 2003;48:2246–55. 12905479 10.1002/art.11073

[2] Mayes MD. Scleroderma epidemiology. Rheum Dis Clin North Am 2003;29:239–54. 12841293 10.1016/s0889-857x(03)00022-x

[3] Silver RM. Clinical aspects of systemic sclerosis (scleroderma). Ann Rheum Dis 1991;50: (suppl): 854–61. 1750797 10.1136/ard.50.suppl_4.854 PMC1033320

[4] LeRoy EC Black C Fleischmajer R. Scleroderma (systemic sclerosis): classification, subsets and pathogenesis. J Rheumatol 1988;15:202–5. 3361530

[5] Pattanaik D Brown M Postlethwaite BC Postlethwaite AE. Pathogenesis of systemic sclerosis. Front Immunol 2015;6:272. 26106387 10.3389/fimmu.2015.00272 PMC4459100

[6] Fleming JN Nash RA Mahoney WM Jr Schwartz SM. Is scleroderma a vasculopathy? Curr Rheumatol Rep 2009;11:103–10. 19296882 10.1007/s11926-009-0015-3 PMC4091992

[7] Solomon JJ Olson AL Fischer A Bull T Brown KK Raghu G. Scleroderma lung disease. Eur Respir Rev 2013;22:06–19. 10.1183/09059180.00005512 PMC4103193 23457159

[8] Razykov I Thombs BD Hudson M Bassel M Baron M. Canadian Scleroderma Research Group. Prevalence and clinical correlates of pruritus in patients with systemic sclerosis. Arthritis Rheum 2009;61:1765–70. 19950328 10.1002/art.25010

[9] El-Baalbaki G Razykov I Hudson M. Association of pruritus with quality of life and disability in systemic sclerosis. Arthritis Care Res 2010;62:1489–95. 10.1002/acr.20257 20506531

[10] Racine M Hudson M Baron M Nielson WR. Canadian Scleroderma Research Group. The impact of pain and itch on functioning and health-related quality of life in systemic sclerosis: an exploratory study. J Pain Symptom Manage 2016;52:43–53. 26876159 10.1016/j.jpainsymman.2015.12.314

[11] Mayes MD. The Scleroderma Book: A Guide for Patients and Families. Oxford: Oxford University Press; 1999.

[12] Johns Hopkins Scleroderma Center. Scleroderma patient stories; 2010. Available at: http://scleroderma.jhmi.edu/patients/patient-stories.html. Accessed 8 September 2021

[13] Razykov I Levis B Hudson M Baron M Thombs BD. Canadian Scleroderma Research Group. Prevalence and clinical correlates of pruritus in patients with systemic sclerosis: an updated analysis of 959 patients. Rheumatology (Oxford) 2013;52:2056–61. 23946437 10.1093/rheumatology/ket275

[14] Théréné C Brenaut E Sonbol H. Itch and systemic sclerosis: frequency, clinical characteristics and consequences. Br J Dermatol 2017;176:1392–3. 27548759 10.1111/bjd.14998

[15] Kim HJ. Pruritus in autoimmune connective tissue diseases. Ann Transl Med 2021;9:441. 33842662 10.21037/atm-20-4894 PMC8033328

[16] Frech TM Baron M. Understanding itch in systemic sclerosis in order to improve patient quality of life. Clin Exp Rheumatol 2013;31: (2 suppl 76): 81–8. 23910614

[17] Friedman JD Dello Buono FA. Opioid antagonists in the treatment of opioid-induced constipation and pruritus. Ann Pharmacother 2001;35:85–91. 11197589 10.1345/aph.10121

[18] Cheng B Liu HW Fu XB. Update on pruritic mechanisms of hypertrophic scars in postburn patients: the potential role of opioids and their receptors. J Burn Care Res 2011;32:e118–25. 21747328 10.1097/BCR.0b013e3182223c32

[19] Salgado RM Alcántara L Mendoza-Rodríguez CA. Post-burn hypertrophic scars are characterized by high levels of IL-1beta mRNA and protein and TNF-alpha type I receptors. Burns 2012;38:668–76. 22226222 10.1016/j.burns.2011.12.012

[20] Frech T Novak K Revelo MP. Low-dose naltrexone for pruritus in systemic sclerosis. Int J Rheumatol 2011;2011:804296. 21918649 10.1155/2011/804296 PMC3171757

[21] Elman S Hynan LS Gabriel V Mayo MJ. The 5-D itch scale: a new measure of pruritus. Br J Dermatol 2010;162:587–93. 19995367 10.1111/j.1365-2133.2009.09586.x PMC2875190

[22] Reich A Szepietowski JC. Non-analgesic effects of opioids: peripheral opioid receptors as promising targets for future anti-pruritic therapies. Curr Pharm Des 2012;18:6021–4. 22747545 10.2174/138161212803582405

[23] Hutchinson MR Zhang Y Brown K. Non-stereoselective reversal of neuropathic pain by naloxone and naltrexone: involvement of toll-like receptor 4 (TLR4). Eur J Neurosci 2008;28:20–9. 18662331 10.1111/j.1460-9568.2008.06321.x PMC2588470

[24] Ekelem C Juhasz M Khera P Mesinkovska NA. Utility of Naltrexone treatment for chronic inflammatory dermatologic conditions: a systematic review. JAMA Dermatol 2019;155:229–36. 30484835 10.1001/jamadermatol.2018.4093

[25] Jones EA Neuberger J Bergasa NV. Opiate antagonist therapy for the pruritus of cholestasis: the avoidance of opioid withdrawal-like reactions. QJM 2002;95:547–52. 12145394 10.1093/qjmed/95.8.547

[26] Smith JP Stock H Bingaman S Mauger D Rogosnitzky M Zagon IS. Low-dose naltrexone therapy improves active Crohn's disease. Am J Gastroenterol 2007;102:820–8. 17222320 10.1111/j.1572-0241.2007.01045.x

[27] Sharafaddinzadeh N Moghtaderi A Kashipazha D Majdinasab N Shalbafan B. The effect of low-dose naltrexone on quality of life of patients with multiple sclerosis: a randomized placebo-controlled trial. Mult Scler 2010;16:964–9. 20534644 10.1177/1352458510366857

